# Selection of Die Material and Its Impact on the Multi-Material Extrusion of Bimetallic AZ31B–Ti6Al4V Components for Aeronautical Applications

**DOI:** 10.3390/ma14247568

**Published:** 2021-12-09

**Authors:** Daniel Fernández, Alvaro Rodríguez-Prieto, Ana María Camacho

**Affiliations:** Department of Manufacturing Engineering, Universidad Nacional de Educación a Distancia (UNED), 28040 Madrid, Spain; alvaro.rodriguez@ind.uned.es (A.R.-P.); amcamacho@ind.uned.es (A.M.C.)

**Keywords:** bimetallic extrusion, multi-material, Ti6Al4V, AZ31B, die material, force, induced damage, wear

## Abstract

This paper investigates the effect that the selection of the die material generates on the extrusion process of bimetallic cylindrical billets combining a magnesium alloy core (AZ31B) and a titanium alloy sleeve (Ti6Al4V) of interest in aeronautical applications. A robust finite element model is developed to analyze the variation in the extrusion force, damage distribution, and wear using different die materials. The results show that die material is a key factor to be taken into account in multi-material extrusion processes. The die material selection can cause variations in the extrusion force from 8% up to 15%, changing the effect of the extrusion parameters, for example, optimum die semi-angle. Damage distribution in the extrudate is also affected by die material, mainly in the core. Lastly, die wear is the most affected parameter due to the different hardness of the materials, as well as due to the variations in the normal pressure and sliding velocity, finding critical values in the friction coefficient for which the die cannot be used for more than one forming stage because of the heavy wear suffered. These results can potentially be used to improve the efficiency of this kind of extrusion process and the quality of the extruded part that, along with the use of lightweight materials, can contribute to sustainable production approaches.

## 1. Introduction

Multi-material applications for the production of structural elements have gained relevance during recent years thanks to equal or increased performance and lower weight compared to their equivalent single material. Working with multiple materials allows designers to combine the mechanical properties of each material to the specific in-service requirements of the component. A reduction in the weight of the final parts is one of the most relevant characteristics when using these advanced materials, which is a key factor in industries such as aerospace to increase the payload in aircrafts and satellites, saving fuel and reducing the environmental impact.

There are several multi-material manufacturing applications; the most extended in aerospace industry is the use of composite material, with both a thermoplastic and a thermosetting matrix. Moreover, in recent years, additive manufacturing has been developed to reduce tooling costs and manufacture parts with shapes that cannot be achieved using conventional methods. Unfortunately, there are still limitations in the use of these multi-material manufacturing methods such as the high temperature needed for composites and the poor surface finishing of additive manufacturing, as well as the low mechanical properties and lack of airworthiness certifications to be used as primary structural parts in aircrafts.

There are several studies about multi-material formation such as Camacho et al. [1] analyzing the effect of shape factors and different assembly fit tolerances on the ductile damage and the microstructure resultant in minting of bimetallic cylinders made of a brass UNS C38500 ring and aluminum alloy UNS A92011 center. Additionally, Zhang et al. [2] used hot rolling to manufacture an Al/Mg/Al composite with a trilaminate structure. Alcaraz and Sevillano [3] used finite element (FE) calculations to study the influence of some extrusion variables such as yield stress ratio between the two materials, location of the layers, thickness ratio, and die angle in the bimetallic tubes composed of two different alloys. Chenot et al. [4] analyzed several methods for the multi-body contact problem such as classical multi mesh approach, single-mesh technique, and Euler formulation, reviewing mechanical and numerical formulations and evaluating the elapsed CPU time for each method.

As mentioned above, bimetals allow combining the properties of dissimilar materials. Magnesium alloys have special interest in the aerospace industry due to their high strength-to-weight ratio, but they also present poor corrosion resistance which is a clear limitation for their in-service performance, as stated by Mordike et al. [5]. Titanium alloys are also widely used in aerospace applications because of their high strength-to-weight ratio, great toughness, fatigue resistance at high temperatures, and good resistance to corrosive environments; however, due to the high flow stress at elevated temperatures, these alloys are difficult to fabricate [6,7]. A common process typically used to obtain multi-material billets composed of a sleeve and a cylindrical core is coextrusion. This is a complex thermomechanical process due to the combination of plastic deformation and diffusion in the interface of both materials because of the pressure and temperatures generated. In addition, for joining dissimilar materials, this process gets more complicated because of the different flow stress characteristics of the materials. Most of the studies about multi-material processes have been carried out using aluminum alloys; for example, the study performed by Khosravifard et al. [8] proved that interface bonding can be associated with material flow velocity and local interface temperature in the Al/Cu clad bimetal rod extrusion process. Another interesting study combining Al and Cu was that by Lapovok et al. [9] on the inter-diffusion improvement in the manufacturing of Cu/Al using severe plastic deformation (SVD) methods. Berski et al. [10] analyzed the strain–stress state in bimetallic rods composed of Cu–Al using two different extrusion ratios in a conical die and in double reduction die. Kocich et al. [11] used the method of twist channel angular pressing (TCAP) to analyze the deformation behavior of an Al/Cu clad composite.

Regarding magnesium alloys in multi-material forming processes, Negendanka et al. [12] analyzed the influence of the die angle and different combining techniques of Mg-core and Al-sleeve on the diffusion layer formation. An analysis of the effect of the extrusion ratio while keeping temperature and ram speed constant in a ZM21 magnesium alloy/CP aluminum coextrusion process was performed by Thirumurugan et al. [13]. Gall et al. [14] proposed a finite element method (FEM) simulation together with experiments to study the behavior of bimetallic Al–Mg billets during the coextrusion process. Rong et al. [15] analyzed the effects on microstructure and mechanical properties of an Mg–Gd–Zn–Zr alloy manufactured by differential thermal extrusion. Moreover, some studies using the hydrostatic extrusion process to obtain bimetallic rods have been performed; Osakada et al. [16] carried out an experimental study using Cu–Al composite rods, determining that uniform deformation occurred for low extrusion ratios, whereas copper rod failed by tension at high extrusion ratios. Moreover, the critical extrusion ratio increased as the die angle decreased. Another study using hydrostatic extrusion is that by Lehmann et al. [17] who analyzed the mechanical strength and fracture properties of hydrostatic coextruded Al–Mg compounds. Nevertheless, there are not too many studies combining different metallic alloys such as titanium and magnesium alloys in terms of density, tensile strength, yield strength, and elastic modulus. Behrens et al. [18] performed a lateral angular coextrusion (LACE) to product semifinished products consisting of aluminum and steel, while Fernández et al. [19] analyzed the effect of different extrusion process parameters and determined the most relevant using the Taguchi method. Regarding the LACE process, a very complete study was performed by Thürer et al. [20] to manufacture coaxially reinforced hollow profiles made of aluminum alloy EN AW-6082 and AISI5120 case-hardening steel 20MnCr5, including the results of shear compression test in the hybrid profiles to measure the shear strength of the bonding area.

This study continues the work mentioned by Fernández et al. [19], but taking into account the material of the die as the driven factor to analyze the effect of the remaining extrusion parameters on the extrusion force, damage, and wear distribution.

## 2. Materials and Methods

### 2.1. Materials, Geometrical Dimensions, and Process Parameters

**Figure 1:**
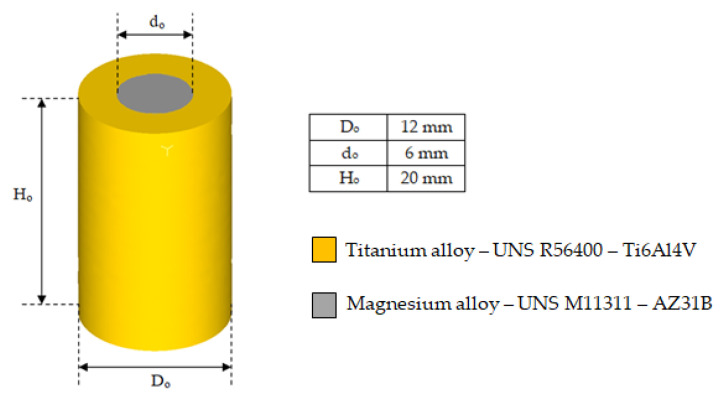
Bimetallic cylinder sketch and initial geometrical dimensions.

The bimetallic cylinders used in the simulations were made of a titanium alloy UNS R56400 sleeve and a magnesium alloy UNS M11211 core (see Figure 1). These two alloys are widely used in aerospace industry due their excellent properties. The most commercial designations for these alloys (Ti6Al4V and AZ31B, respectively) are used henceforth.

**Figure 2:**
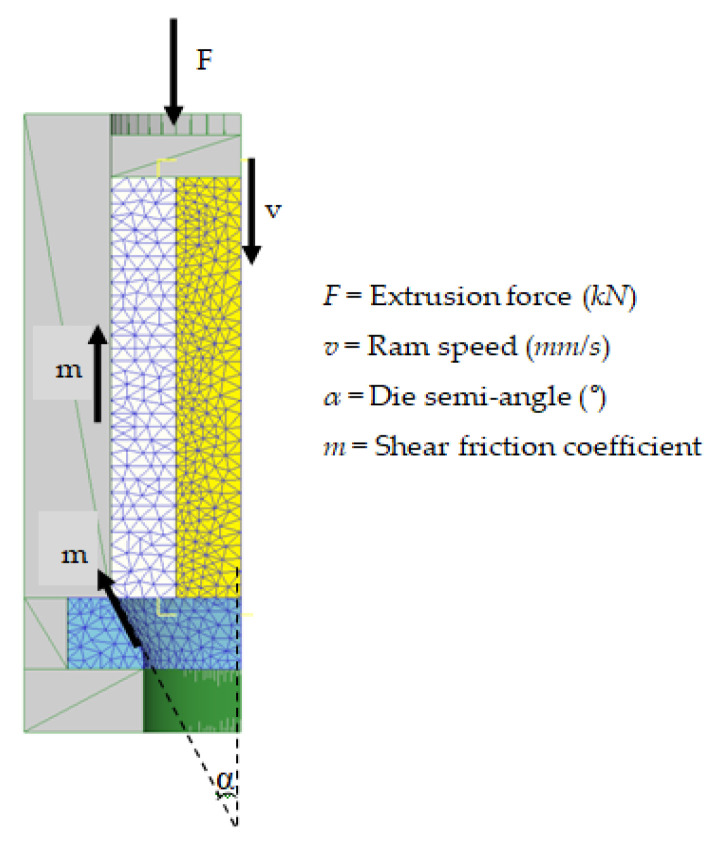
Extrusion process schema.

The parameters of the extrusion process considered in this work are presented in Figure 2.

The chemical compositions were extracted from Daniel et al. [19]; the physical and mechanical properties for AZ31B were obtained from Avedesiam et al. [21], while those for Ti6Al4V were obtained from Donachie [22].

The chemical composition of each steel used to model the die and a comparison of their physical and mechanical properties are listed in Table 1 and Table 2. Physical and mechanical properties were extracted from Davis et al. [23], and chemical compositions of H13, 25CrMo4. 53 HRC, AISI3310, and AISI316 were obtained from [24,25,26,27,28,29], respectively.

Material selection criteria for die components included Young’s modulus, hardness of the workpiece material, and availability on the market.

- AISI316 is one of the most used austenitic stainless steels in the world, showing improvements in the corrosion and high-temperature resistance due to the addition of molybdenum.
- H13 is a chromium hot work tool steel, widely used in hot and cold working applications. This tool steel stands out for its high toughness and fatigue resistance.
- 25CrMo4, also known under the classification AISI4130, is a versatile alloy with good overall combination of strength, toughness, fatigue strength, and corrosion resistance. The normal applications for this steel are fittings with a large cross-section and high-stress working conditions such as a shafts and gear wheels; it was selected for this study due to its low hardness (48 HRC).
- AISI3310 steel is commonly used in components with a large cross-section requiring high toughness and core strength, such as bearings and gear applications. This was the steel with a high percentage of Ni and the highest hardness (58.22 HRC) in the present study.
- AISI52100 (53HRC) is a high-carbon chromium steel. Because of its combination of strength, hardness, and workability, this steel is particularly useful in bearings, mill rolls, and vehicle parts. This steel was chosen in this study for its high modulus of elasticity (210 GPa) staying constant independently of the temperature.

### 2.2. Finite Element Modeling

Finite element simulations were performed using the commercial finite element program DEFORM3D© v11.2 [30].

The ram, container, holder, and blocker (extrusion tooling) were modeled as rigid objects. The bimetallic cylinders were modeled as an assembly between two plastic objects (sleeve and core). The die was modeled as an elastic object.

**Figure 3:**
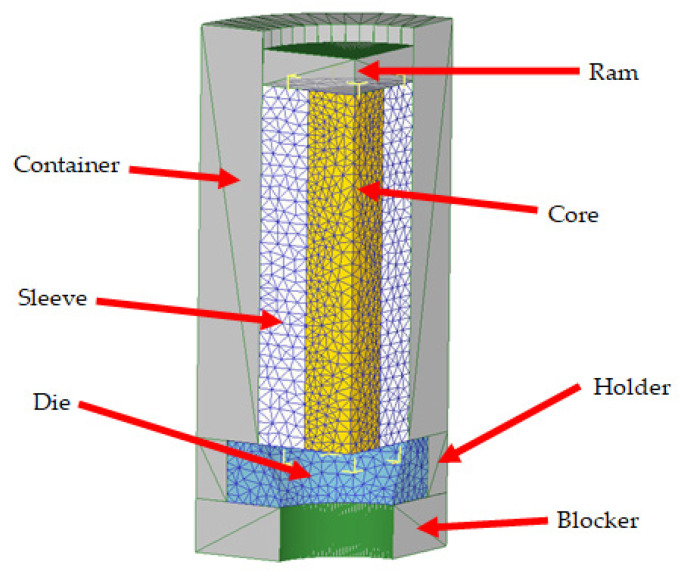
Object set up for simulations.

In order to reduce the computation time and the size of the database files, taking into account the axial symmetry of the coextrusion process, only one-quarter of the problem was modeled. Figure 3 shows the whole setup for the simulation.

Considering the heat transfer across the billet, die, and the extrusion tooling, all parts were meshed with tetrahedral elements. The heat transfer coefficient between sleeve and core and between sleeve and die was set to 11 N/(s·mm·°C), that between extrusion tooling elements and die was set to 5 N/(s·mm·°C), and that between billet/die/tooling and air was set to 0.02 N/(s·mm·°C).

The contact conditions among the different objects were also defined. Rigid and elastic objects were considered “masters” (those that deform) and the plastic objects were considered “slaves” (those that are deformed). In the case of the sleeve and core interaction, where both objects are plastic, the titanium alloy was defined as the “master” and the magnesium alloy was defined as the “slave”; due to the assumption that the fitting between these two parts was performed with interference, the friction took the maximum value of 1. In addition, sticking and non-separable conditions were also defined for these objects.

Regarding the die, to avoid movement with respect to the rigid parts, the sticking condition was also defined.

The friction model used in the simulations was the shear model as the range of friction coefficients used in this study was from 0.1 to 0.7; according to Rowe [31] and Leu et al. [32], the upper limit of the Coulomb friction coefficient is 0.577 for von Mises and 0.5 for Tresca criteria. Moreover, this friction model is typically used in metal formation analysis. Materials AZ31B, Ti6Al4V, and die steels were assumed to be isotropic throughout the process. 

The normalized Cockcroft and Latham criterion [33] was used to evaluate the damage factor on the extrudate. This method is widely used to predict the damage, mainly in cold formation processes, because of its simplicity and very little material data required for calculations. This criterion is based on the hypothesis that the accumulation of damages occurs only when at least one of the principal stress components is tensile, as represented in Equation (1).

(1) $$\int_{0}^{\varepsilon_{f}}\left(\frac{\sigma_{max}}{\sigma_{H}}\right)d\varepsilon =C,$$

where *ε* is the equivalent plastic strain, *ε_f_* is the limit fracture strain, *σ_max_* is the maximum principal stress, *σ_H_* is the stress according to the Huber–Mises hypothesis, and *C* is the parameter that is usually called the material constant, and its value is experimental determined.

Damage is associated with stress states, mainly because of hydrostatic stress; according to the hydrostatic stress criterion (HSC), “whenever hydrostatic stress at a point on the center line in the deformation zone becomes zero and it is compressive elsewhere, there is a fracture initiation leading to central burst” [34,35,36].

The calculus of the wear in the die was performed using Archard’s wear model [37,38,39]. In this model, the wear depth is directly proportional to the wear coefficient (*K*), the interface pressure (*P*), and the sliding velocity (*v*) between die and billet, and it is inversely proportional to the die hardness (*H*), according to Equation (2).

(2) $$W=\int^{\;}K\cdot \;\frac{p^{a}\cdot v^{b}}{H^{c}}\cdot dt,$$

where *a*, *b*, *c*, and *K* are experimentally calibrated coefficients; *a*, *b* are commonly taken as 1, and *c* = 2 for tool steels. K was taken as 2 × 10^−5^.

However, die material behavior (such as hardness and wear coefficient) will change significantly with varying temperature over 400 °C. Therefore, in order to avoid this effect and to use modified Archard’s wear theory to take into account the variations in the values of K and hardness with the temperature, the maximum working temperature used in this study was 300 °C.

### 2.3. Methodology

The aim of this study was to establish if the material of the die significantly affects to the extrusion force and damage factor values, as well as if their behavior with the different extrusion parameters changes with the material of the die.

The parameters affecting the extrusion process taken into account for this study were the following:

- Ram speed (mm/s) and temperature (°C) as process parameters.
- Die semi-angle (°), shear friction factor, and extrusion ratio (*A*_0_/*A_f_*) as tool parameters.
- Shape factor (*H*_0_/*D*_0_) and diameter ratio (*D*_0_/*d*_0_) as geometric parameters.
- Die material.

*H*_0_ is the initial height of the billet, *D*_0_ is the initial external diameter of the sleeve, *d*_0_ is the initial diameter of the core, and *A*_0_ and *A_f_* are the initial and final area, respectively, of the cross-section of the billet.

The behavior of die wear has not yet been investigated during a multi-material extrusion process. As modeling of the wear was done according to Archard’s model, the parameters taken into account were ram speed, temperature, and friction.

Ram speed may influence the sliding velocity, which is a factor to calculate wear according to Archard’s model; furthermore, an excessive ram speed may cause surface defects to the extrudate such as over-burning, adhesion, and gravure.

Temperature is not in Archard’s wear model [38,39]. The range of temperature considered in this study was below 400 °C; therefore, the friction coefficient and the hardness would remain unaffected. However, the temperature has a direct influence on the metal plasticity, flow resistance, and flow stress curves, and these factors may impact in the die wear because they may affect the normal pressure and sliding velocity.

Although Archard’s wear model does not include direct friction-related factors, the friction parameter has an influence on the material flow and stress distribution of the extrusion die, which may have certain effects on the wear factor.

The design of experiments (DOE) to evaluate the effect of each parameter on the extrusion force and damage factor is shown in Table 3 for the range of extrusion parameters taken in the simulations and in Table 4 for the baseline values of the simulations.

The DOE methodology in this study consisted of varying the parameter to be studied while holding the remainder to their baseline values. Each set of simulations for each parameter was repeated while changing the die material to analyze the behavior of the extrusion force and damage factor with respect to each parameter.

The methodology to evaluate the wear on the die was the same but only using ram speed, temperature, and friction as parameters of the study.

## 3. Results and Discussion

This section is split into three parts covering the effects of the extrusion parameters for each die material on the extrusion force, damage factor, and die wear.

### 3.1. Effects of the Extrusion Parameters on the Extrusion Force and FE Model Validation

**Figure 4:**
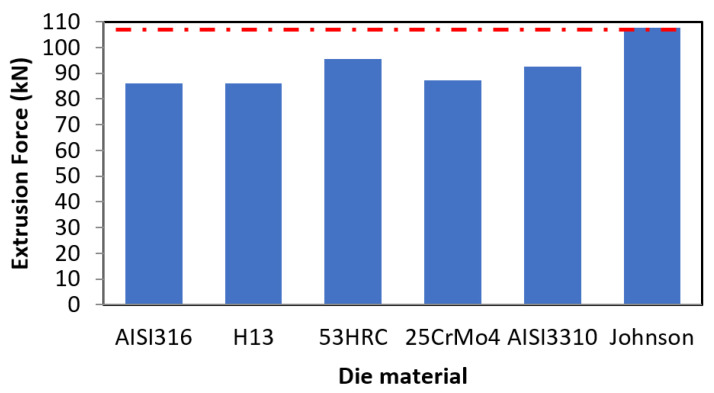
Extrusion force comparison among the different die materials for the baseline conditions and comparison with semi-empirical method of Johnson for FE model validation.

After performing the simulations according to the methodology defined in Section 2.3, the results obtained for each parameter in the baseline are shown in Figure 4.

A difference of 9.59 kN can be observed between the highest (53HRC) and the lowest (AISI316) value of the extrusion force, which represents an increase of 11%.

In Figure 4, results from the semiempirical model of Johnson, described in the work of García et al. [40], were included for FE model validation. This method is commonly used as a reference in the analysis of extrusion processes and typically used as an upper limit of the extrusion force in direct extrusion. To this aim, the average yield stress was calculated considering the volume fraction of the AZ31B core and Ti6Al4V ring, proven as valid in the work of Gisbert et al. [41].

The sections below show the effect of the variation of the process parameters on the extrusion force, analyzing if the behavior is the same independent of the die material, as well as the variation in the values of the extrusion force for each material.

#### 3.1.1. Core Diameter

**Figure 5:**
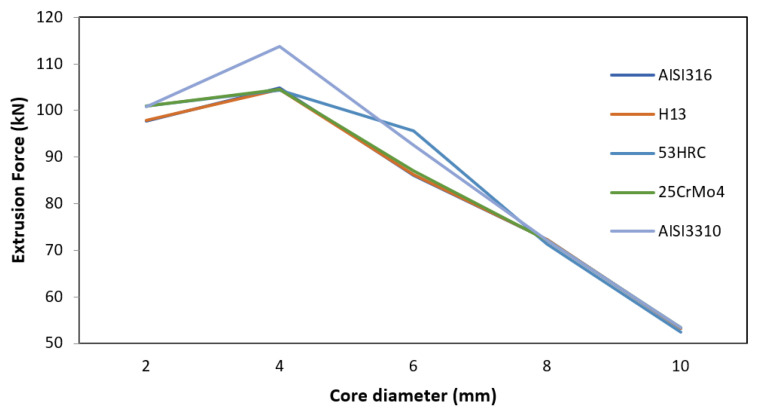
Effect of core diameter on the extrusion force for different die materials.

A peak can be observed for the core diameter value of 4 mm; however, after this point, the values of the extrusion force decreased as the core diameter increased, converging to the values of the extrusion force for the different die materials with 8 mm core diameter. Therefore, the effect of this parameter was inversely proportional to the necessary extrusion force for all die materials, as shown in Figure 5.

The maximum difference in the extrusion force among die materials occurred at the baseline. This can be explained by the reduction in volume of Ti6Al4V due to the increment in core diameter. As more force is needed to extrude Ti6Al4V than AZ31B due to the flow curves of each material, if the volume of Ti6Al4V is lower, the force needed will also be lower.

#### 3.1.2. Billet Height

The behavior of the extrusion force with the billet height was completely the opposite with respect to the core diameter. As the billet height increased, the extrusion force increased.

**Figure 6:**
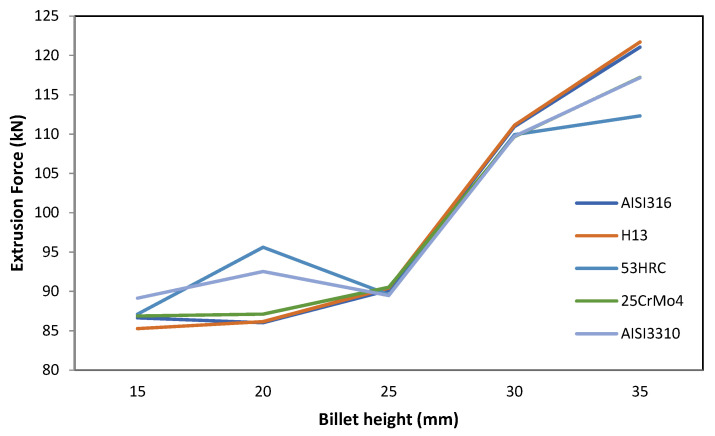
Effect of billet height on the extrusion force for different die materials.

It can be observed in Figure 6 that the biggest increment in extrusion force, measured by the slope of the line, occurred between 25 and 30 mm of billet height (shape factor between 2 and 2.5).

This is because, as the height increases, the contact area of the billet with the container also increases; this causes an increase in the energy component due to friction and, consequently, an increase in the total force required.

#### 3.1.3. Friction

**Figure 7:**
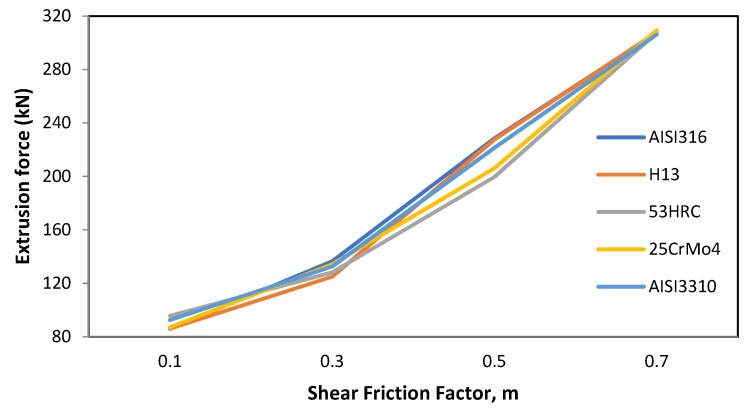
Effect of friction variation on the extrusion force for different die materials.

This parameter had the biggest impact on the extrusion force, going from 86.02 kN for the lowest friction factor to 309.40 kN for the highest one. The behavior was similar to the billet height, and the biggest difference in extrusion force among die materials was 28.93 kN (shear friction factor of 0.5) for AISI316 and 53HRC, as shown in Figure 7.

#### 3.1.4. Extrusion Ratio

The extrusion ratio affects the final cross-section of the billet and, therefore, the shape factor. The expected result is an increase in the extrusion force with the extrusion ratio because, to get a smaller cross-section using the same semi-die angle, the distance the billet has to travel through the die is larger; thus, as with billet height, an increase in the contact area increases causes the component due to friction to increase, along with a logical increment in the extrusion force.

**Figure 8:**
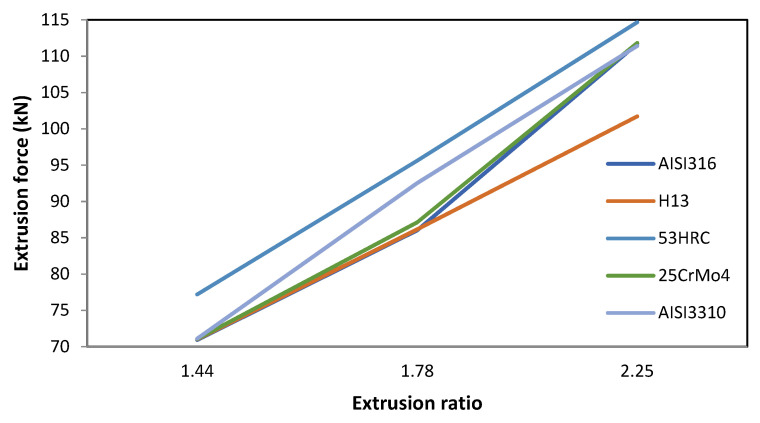
Effect of extrusion ratio variation on the extrusion force for different die materials.

Figure 8 confirms this expectation and highlights the directly proportional relationship between extrusion force and extrusion ratio.

In this case, the biggest difference in the extrusion force with the different die materials occurred at an extrusion ratio of 2.25, with 12.97 kN between 53HRC and H13. In this case, the extrusion force showed an important dependence on the die material.

#### 3.1.5. Ram Speed

The effect of this parameter on the extrusion force is inversely proportional to the extrusion force. This phenomenon occurs because, as the ram speed increases, it also increases the temperature due to the friction in the interface die/billet; accordingly, the stress necessary to deform the billet and, thus, the extrusion force will be lower.

For ram speeds higher than 3 mm/s, the variation in the extrusion force tended to be lower than 2 kN. This is due to the compensation for the increment in temperature, which reduces the stress necessary to achieve a certain strain, and the rise in strain rate involves a higher stress to deform the material.

**Figure 9:**
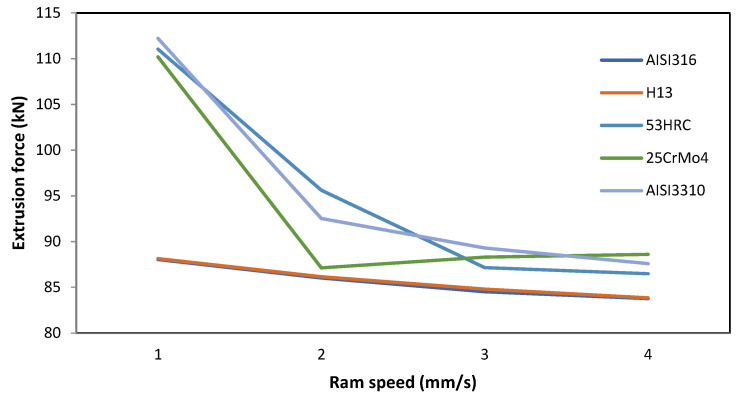
Effect of ram speed variation on the extrusion force for different die materials.

Figure 9 also shows that the biggest difference in extrusion force among die materials occurred for the lowest value of the ram speed (1 mm/s) between AISI3310 and AISI316.

#### 3.1.6. Temperature

The effect of this parameter is similar to that produced by the core diameter and the ram speed. Normally, as the temperature increases, the stress necessary to deform a part is lower in line with material flow stresses.

**Figure 10:**
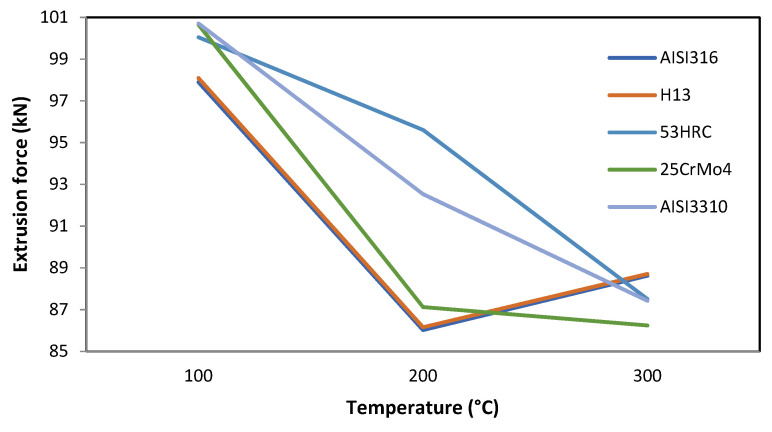
Effect of temperature variation on the extrusion force for different die materials.

This effect can be observed in Figure 10, where the biggest difference in the extrusion force values among the different die materials was produced at the baseline value of 200 °C.

Another important phenomenon is that, for the AISI316 and H13 die materials, the extrusion forces increased again at temperatures higher than 200 °C.

#### 3.1.7. Die Semi-Angle

This parameter is most affected by the die material. In extrusion, there is a semi-angle at which the extrusion force needed for the process is the minimum for the same extrusion ratio. This is called the optimum die semi-angle. Thus, at lower or higher semi-angles, the extrusion force will be higher.

This is a matter of balance between the area of contact and the abrupt change in cross-section and, therefore, energy contributions due to friction and internal distortion. For low semi-angles, the area of contact to achieve a certain reduction in area is larger but the change in cross-section occurs more gradually. For higher semi-angles, the opposite is true, producing an abrupt change in the cross-section for a very short distance traveled over the die.

**Figure 11:**
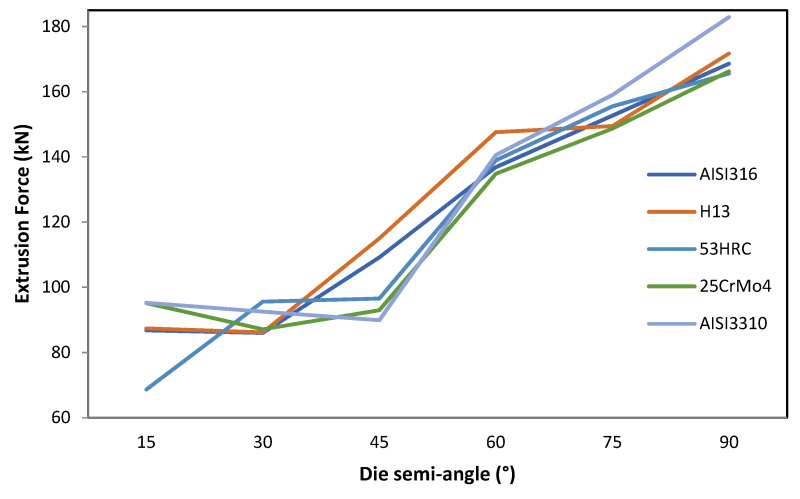
Effect of die semi-angle variation on the extrusion force for different die materials.

Figure 11 shows that the die optimum semi-angle ranged from 15° for 53HRC to 45° for AISI3310.

It is important to note that the optimum die semi-angle value depended on the material of the die. The greatest deviation of the extrusion force occurred for a die semi-angle of 15° between AISI3310 and 53HRC (26.61 kN).

### 3.2. Effects of the Extrusion Parameters on the Damage Factor

Another important aspect that can be analyzed by finite element analysis is the damage induced in the workpiece as a consequence of the formation process. This factor is mainly related to the quality of the extrudate since high values of damage can lead to fracture of the part during the component service.

**Figure 12:**
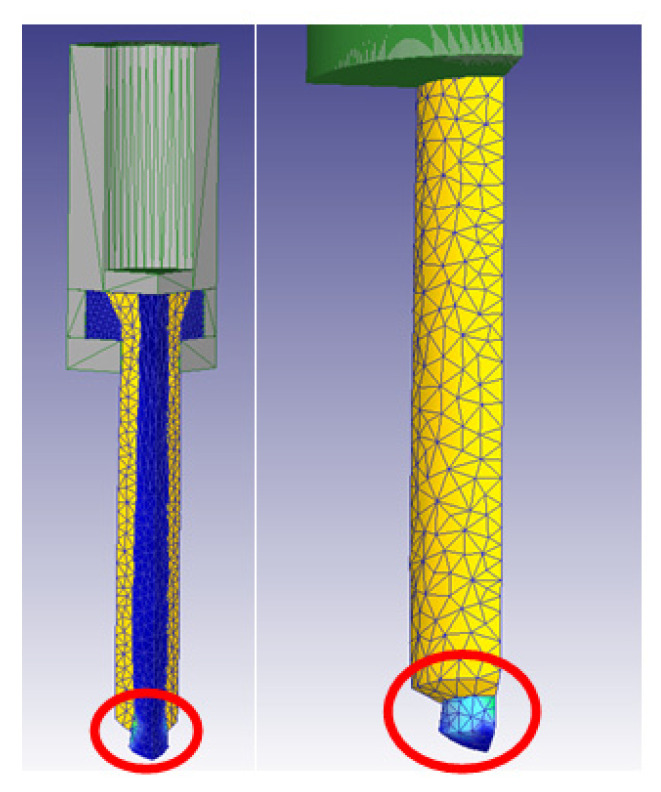
General damage location in the core of the billet.

The study of the effect of the different extrusion parameters on the damage factor focused on the core of the billet. After performing the simulations, the results obtained showed that the damage was spread over the entire surface of the ring, whereas the core damage was mainly centered on the same area, extending to the rest of the core only under certain conditions. This area can be observed in Figure 12.

As all the damage was concentrated in the bottom part of the extrudate, it could be easily removed from the final part. Nevertheless, there were some configurations in which the damage was all over the extrudate. The main parameters mediating this effect were die semi-angle, extrusion ratio, and billet height, whereas parameters such as temperature, ram speed had hardly any influence on the damage.

#### 3.2.1. Die Semi-Angle

**Figure 13:**
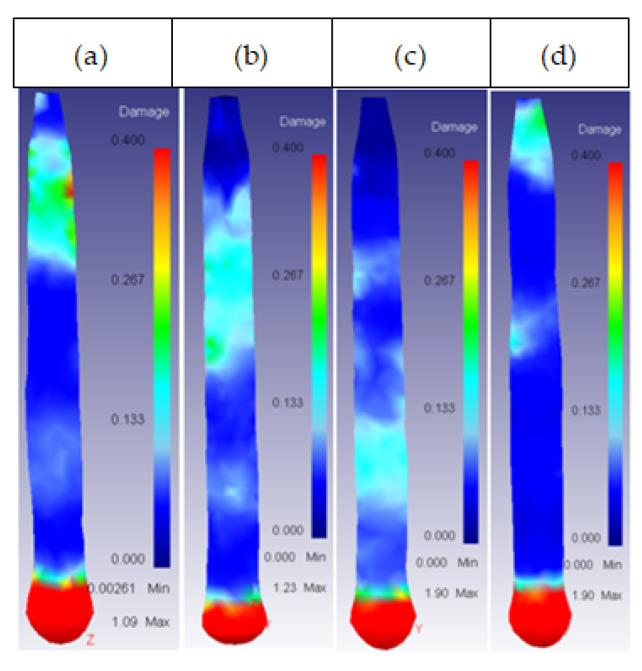
Damage distribution in the core: (**a**) 53HRC α = 75°; (**b**) 25CrMo4 α = 75°; (**c**) AISI3110 α = 75°; (**d**) H13 α = 90°.

**Figure 14:**
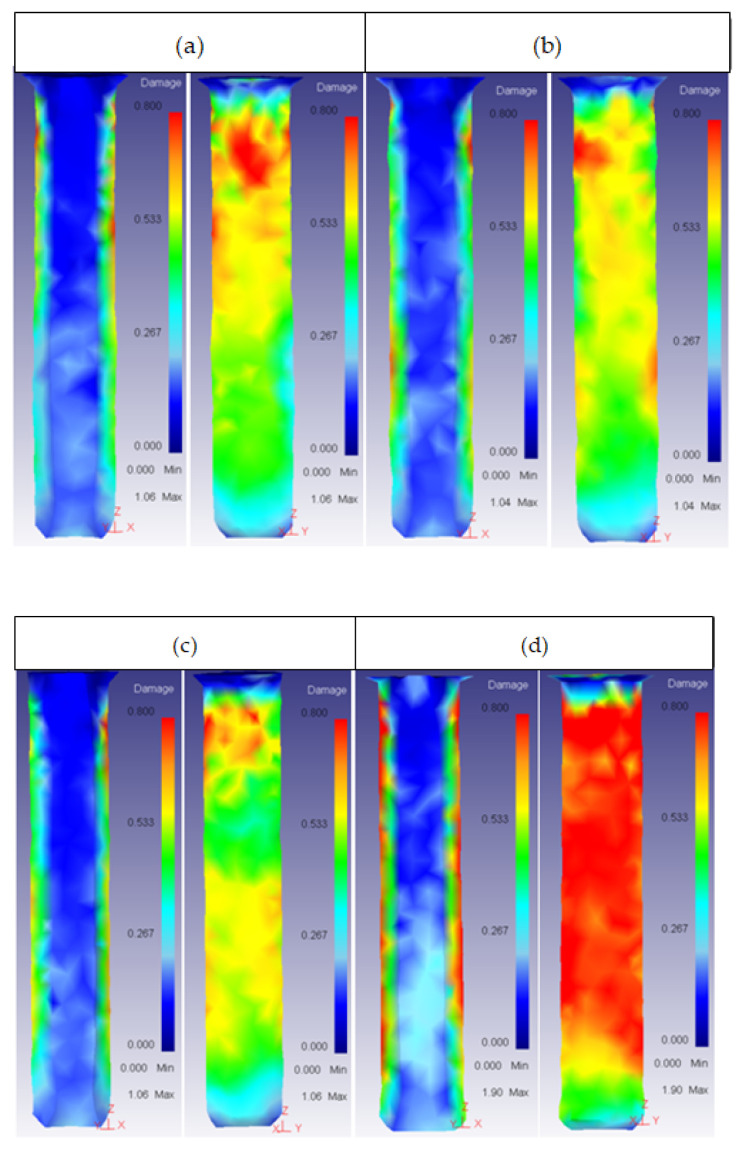

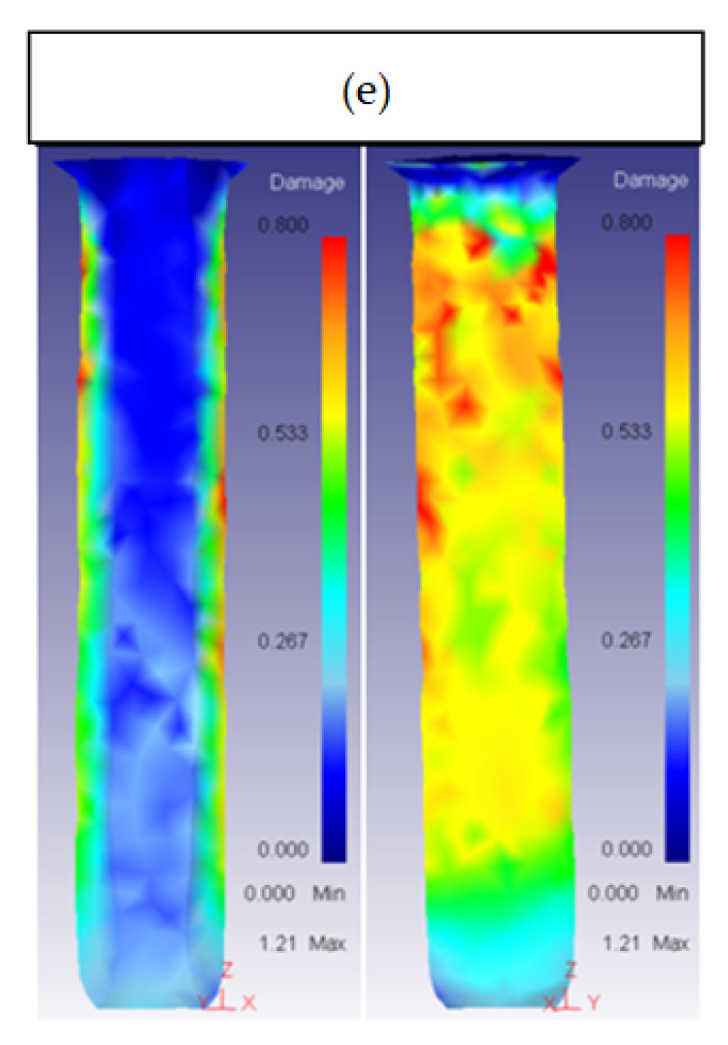
Damage distribution in the ring (inner and outer part): (**a**) 25CrMo4 α = 60°; (**b**) 53 HRC α = 60°; (**c**) AISI316 α = 60°; (**d**) AISI3110 α = 75°; (**e**) H13 α = 60°.

This was the most important parameter regarding the damage factor because there was a semi-angle at which the damage spread through the whole extrudate. This critical semi-angle value varied depending on the die material, as can be observed in Figure 13 and Figure 14 for the core and the ring plus core, respectively.

For angles higher than 60°, the damage started increasing in both the ring and the core, spreading across the extrudate. This could cause a crack, affecting the working life of the part. As the extrusion force also increased from 45° in all die material cases, it is recommended to keep the semi-angle between 15° and 45° to obtain the best results.

Lastly, it is important to realize that the damage growth in the ring was not the same in inner and outer part. The die semi-angle barely affected the damage in the inner part.

#### 3.2.2. Extrusion Ratio

**Figure 15:**
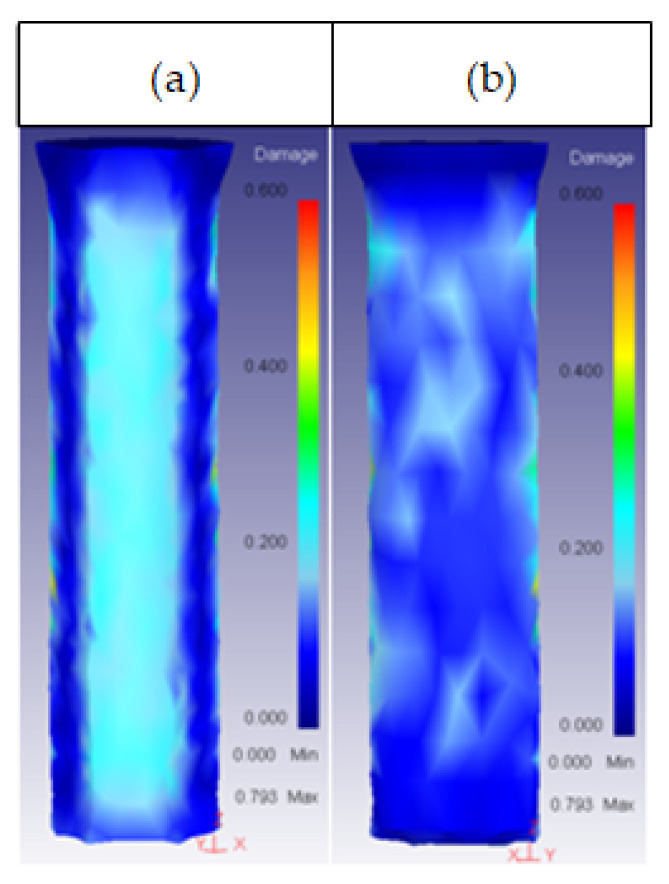
Damage distribution in inner (**a**) and outer (**b**) part of the ring for 53HRC R_E_ = 1.44.

The extrusion ratio had a big influence on the damage factor of the ring. In general, for low values of extrusion ratio (1.44), the damage in the inner face of the ring was highest, as shown in Figure 15.

**Figure 16:**
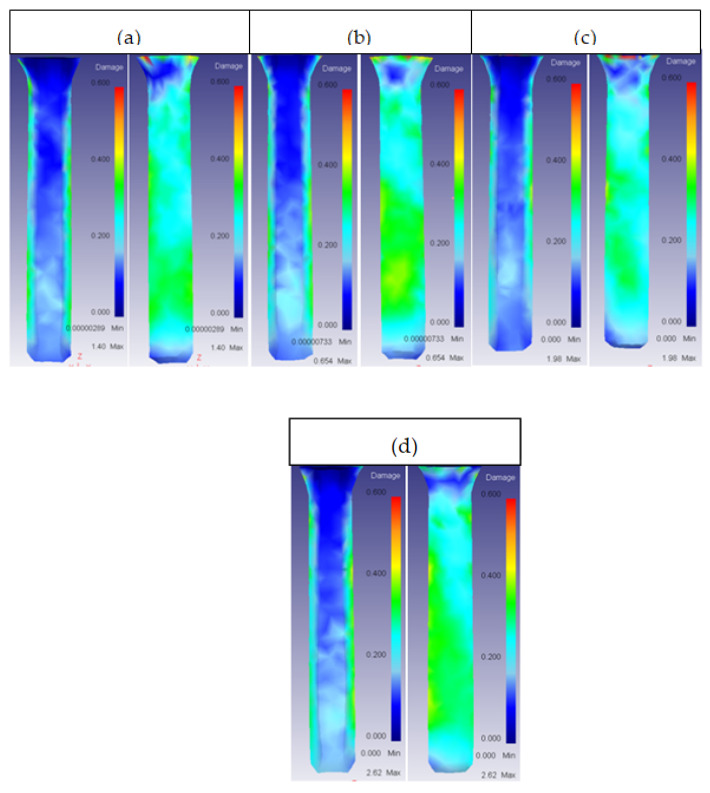
Damage distribution in inner and outer part of the ring: (**a**) 25CrMo4 R_E_ = 2.25; (**b**) 53HRC R_E_ = 2.25; (**c**) AISI3310 R_E_ = 2.25; (**d**) H13 R_E_ = 2.25.

For the outer part of the ring, the effect was opposite, whereby increasing the extrusion ratio increased the damage, as shown in Figure 16.

**Figure 17:**
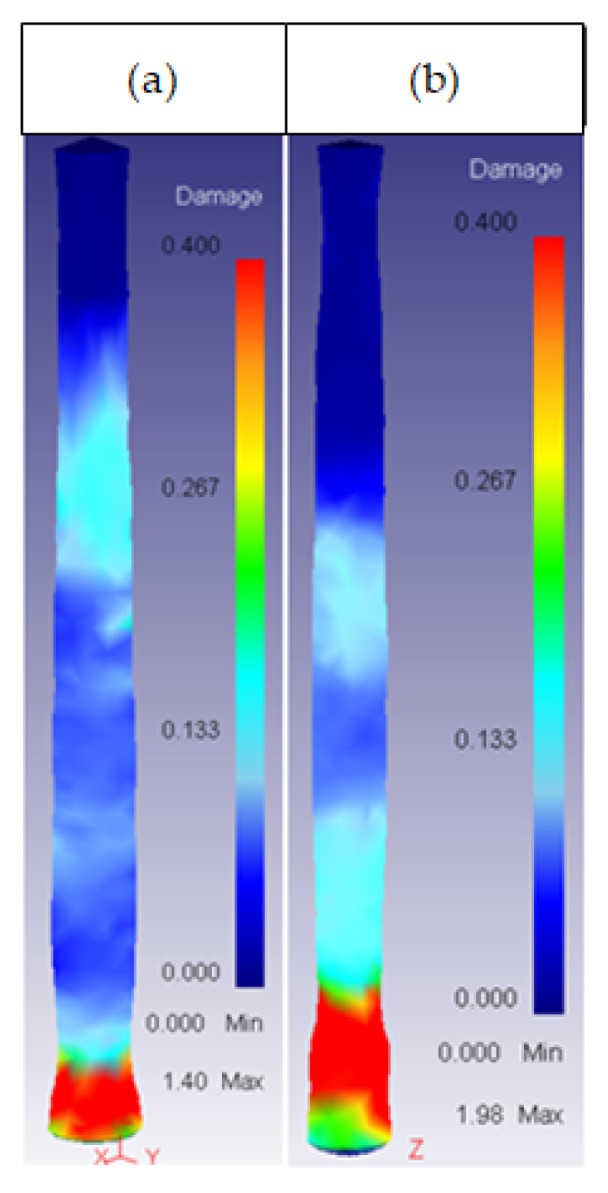
Damage distribution in the core: (**a**) 25CrMo4 R_E_ = 2.25; (**b**) AISI3310 R_E_ = 2.25.

In the core, the damage generally extended across the surface in contact with the ring, as shown in Figure 17.

#### 3.2.3. Billet Height

**Figure 18:**
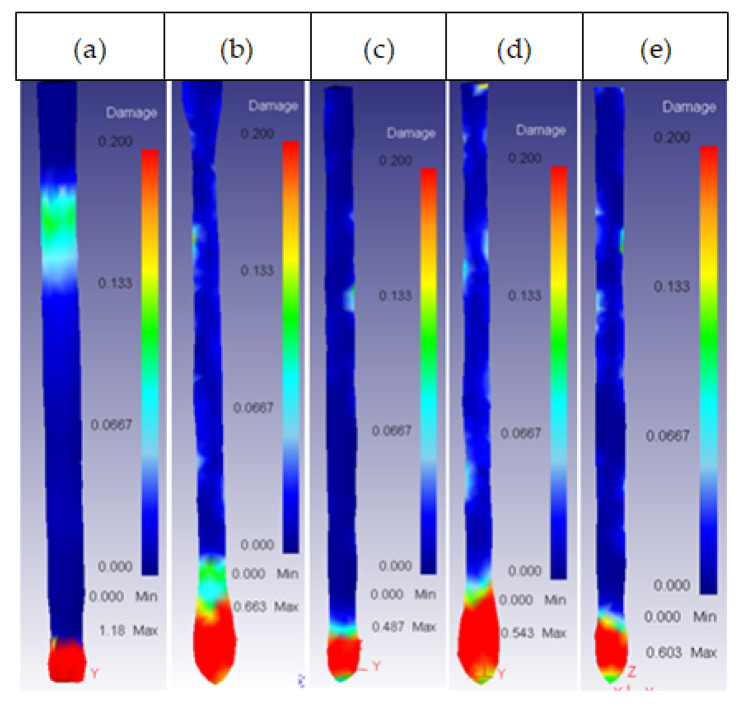
Damage distribution in the core: (**a**) 25CrMo4 H_o_ = 30 mm; (**b**) 53HRC H_o_ = 35 mm; (**c**) AISI316 H_o_ = 35 mm; (**d**) AISI3110 H_o_ = 35 mm; (**e**) H13 H_o_ = 30 mm.

This parameter had an effect on the core damage only for high values of height, with a 2.5 shape factor. At this value, the damage started spreading across the core surface, as shown in Figure 18.

**Figure 19:**
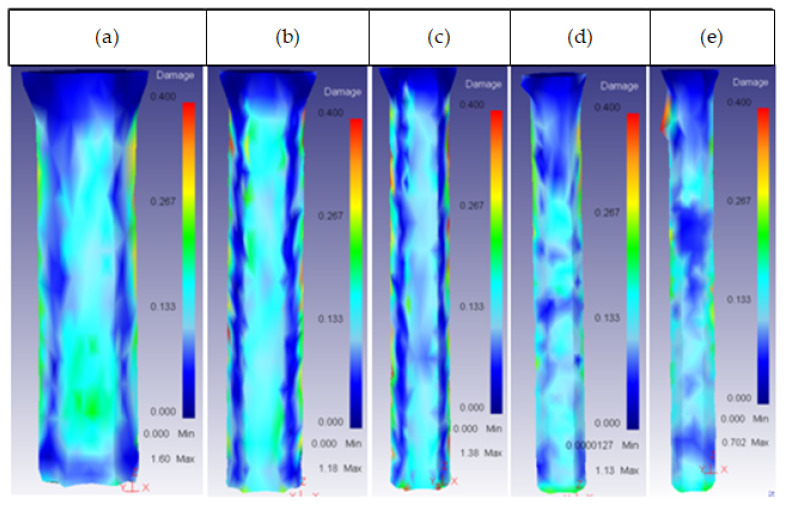
Damage distribution in the inner part of the ring: (**a**) 25CrMo4 H_o_ = 15 mm; (**b**) 25CrMo4 H_o_ = 20 mm; (**c**) 25CrMo4 H_o_ = 25 mm; (**d**) 25CrMo4 H_o_ = 30 mm; (**e**) 25CrMo4 H_o_ = 35 mm.

In the ring, this parameter most affected the inner part, reaching a maximum damage distribution at 20 mm height; from this point, the damage distribution decreased with the increase in height, as shown in Figure 19.

#### 3.2.4. Friction

This parameter only had an effect on the ring part of the billet as it was the only part in direct contact with the container and the die, where the friction factor varied.

**Figure 20:**
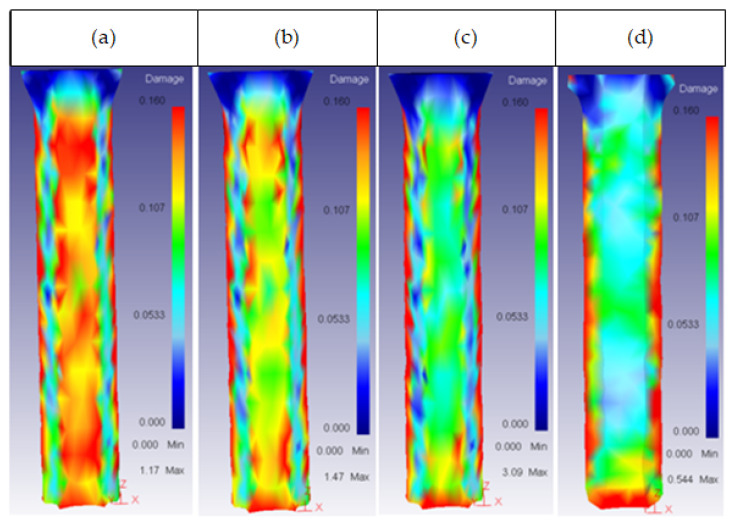
Damage distribution in the inner part of the ring: (**a**) AISI316 *m* = 0.1; (**b**) AISI316 *m* = 0.3; (**c**) AISI316 *m* = 0.5; (**d**) AISI316 *m* = 0.7.

The behavior was the same for all the die materials, producing the maximum damage for *m* = 0.5 on the face of the ring in direct contact with the container and the die, while the damage decreased on the inner face with the increase in friction factor, being highest for *m* = 0.1, as shown in Figure 20.

**Figure 21:**
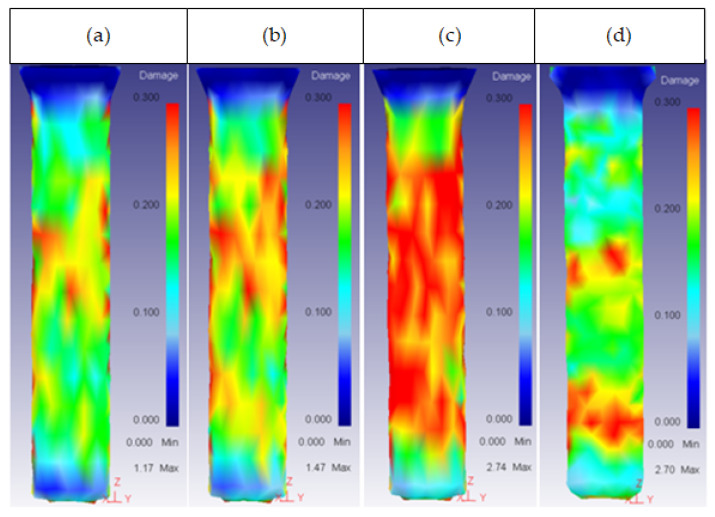
Damage distribution in the outer part of the ring: (**a**) AISI316 *m* = 0.1; (**b**) AISI316 *m* = 0.3; (**c**) AISI316 *m* = 0.5; (**d**) AISI316 *m* = 0.7.

Lastly, for friction factors higher than 0.5, damage in the ring decreased as shown in Figure 21. This behavior may have been caused by the increase in temperature with friction factor, as the damage factor is calculated by measuring stress and the stress decreased with the increase in the temperature according to the flow stress curves.

**Figure 22:**
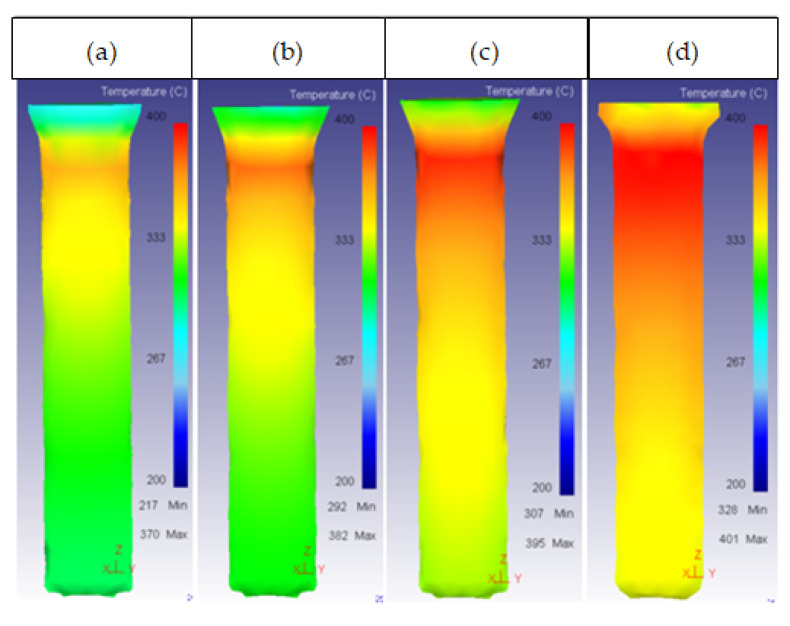
Temperature distribution in the ring: (**a**) AISI316 *m* = 0.1; (**b**) AISI316 *m* = 0.3; (**c**) AISI316 *m* = 0.5; (**d**) AISI316 *m* = 0.7.

Figure 22 shows the variation in temperature with friction factors.

#### 3.2.5. Core Diameter

The effect of this parameter was similar to that of friction, affecting only the ring part of the billet. Both the outer and the inner face showed similar behavior, producing the greatest damage for a core diameter of 10 mm. There was also an optimum value of core diameter, 4 mm, for which the damage distribution was lowest. Therefore, the damage decreased as the core diameter increased until reaching a minimum value, beyond which the damage increased with core diameter.

**Figure 23:**
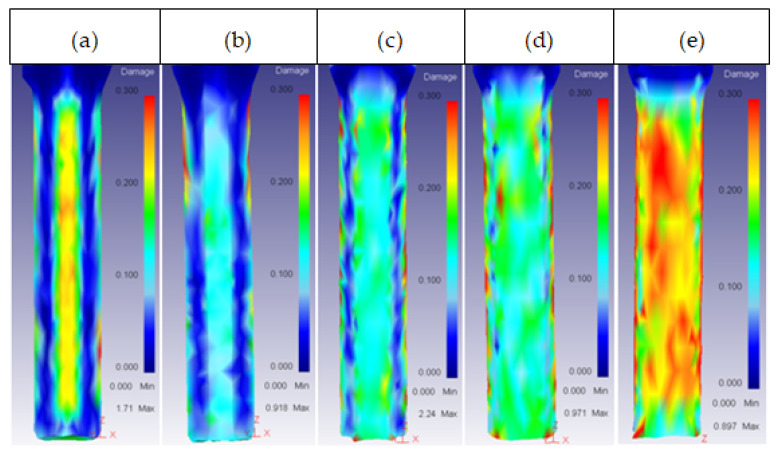
Damage distribution in the inner part of the ring: (**a**) H13 d = 2 mm; (**b**) H13 d = 4 mm; (**c**) H13 d = 6 mm; (**d**) H13 d = 8 mm; (**e**) H13 d = 10 mm.

**Figure 24:**
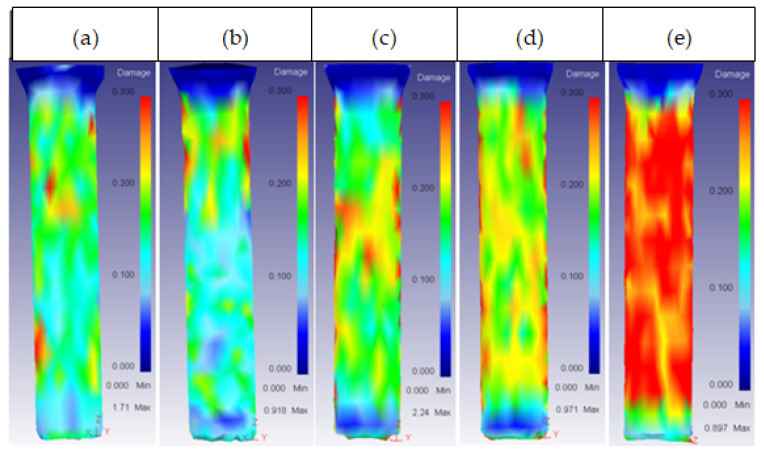
Damage distribution in the outer part of the ring: (**a**) H13 d = 2 mm; (**b**) H13 d = 4 mm; (**c**) H13 d = 6 mm; (**d**) H13 d = 8 mm; (**e**) H13 d = 10 mm.

The main difference in the behavior of the inner and outer face was found for a core diameter value of 2 mm, where the ring showed the second largest damage distribution. All behaviors can be observed in Figure 23 and Figure 24.

This effect was due to the reduction in thickness of the ring. Larger thicknesses denote low values of the core diameter; thus, the contact area with the core is smaller, which causes an increment in the stress. As the core diameter increases, the contact area also increases, but the reduction in thickness causes the ring to receive more damage during the process.

### 3.3. Effects of the Extrusion Parameters on the Die Wear

As explained in Section 2, only ram speed, temperature, and friction were considered as relevant parameters to study the wear on the die.

**Figure 25:**
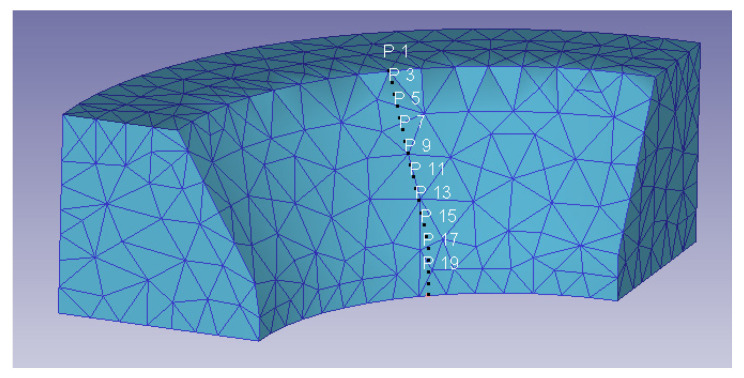
Wear path through the die.

Figure 25 shows the path taken into account to measure the wear variation through the die.

**Figure 26:**
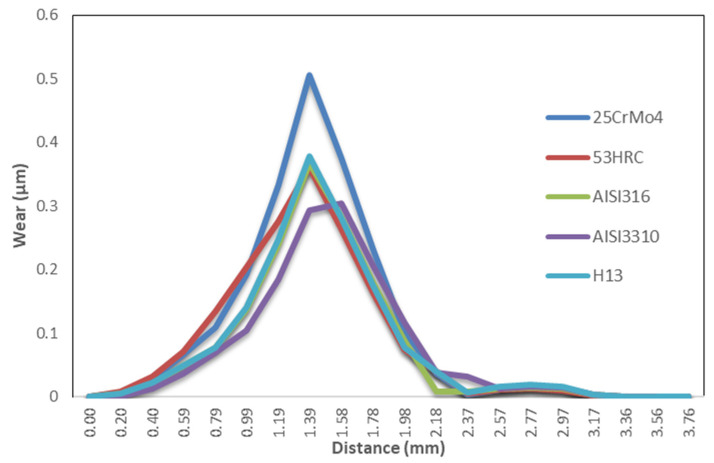
Wear distribution chart through the die for baseline conditions.

**Figure 27:**
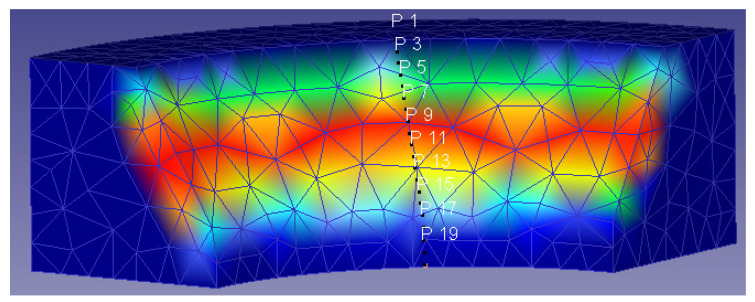
Wear distribution through the die for baseline conditions.

The wear distribution for baseline conditions is presented in Figure 26 and Figure 27.

The first conclusion that can be obtained from the chart is that wear distribution was highly dependent on the hardness, as expected, with the highest wear occurring in the die material with the lowest hardness value (25CrMo4; 48).

Secondly, maximum wear values appeared during the first stages of displacement through the die, reaching a peak before the midpoint, after which the wear gradually decreased until it reached the change in section.

The sections below describe how the wear distribution was affected by different extrusion parameters for different die materials.

#### 3.3.1. Ram Speed

**Figure 28:**
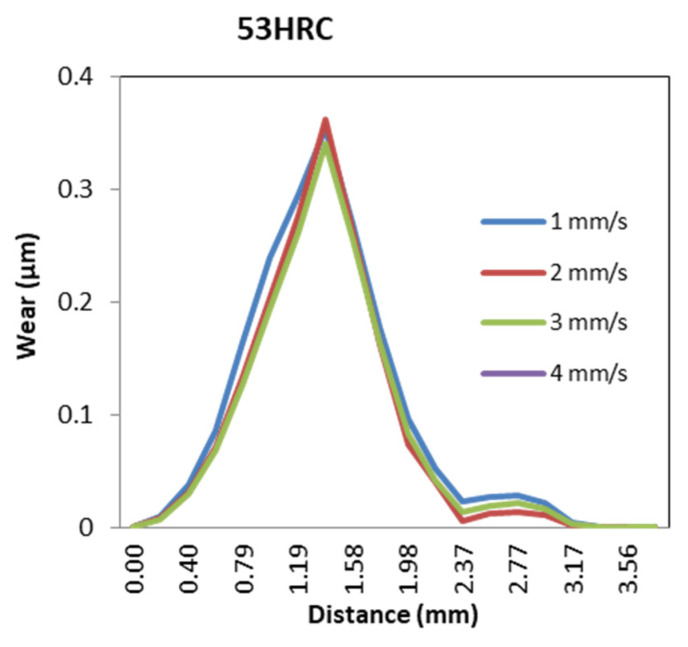

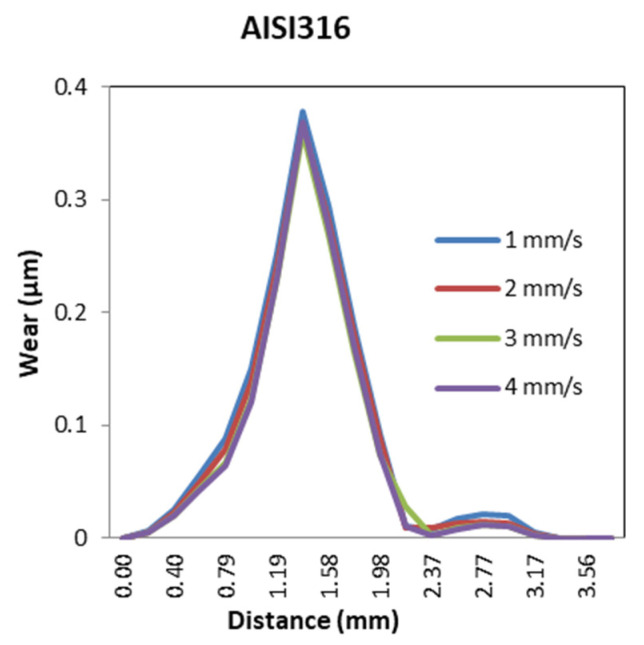
Wear distribution function of ram speed for 53HRC and AISI316 die materials.

Ram speed had almost no impact on the wear distribution for die materials 53HRC and AISI316, as can be observed in Figure 28.

**Figure 29:**
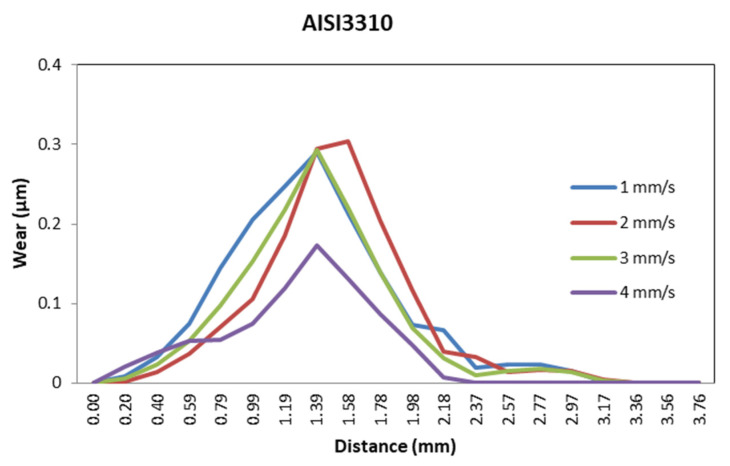
Wear distribution function of ram speed for AISI3310.

The die material most affected by ram speed was AISI3310, which suffered the highest reduction in wear for ram speed values of 4 mm/s, as shown in Figure 29.

**Figure 30:**
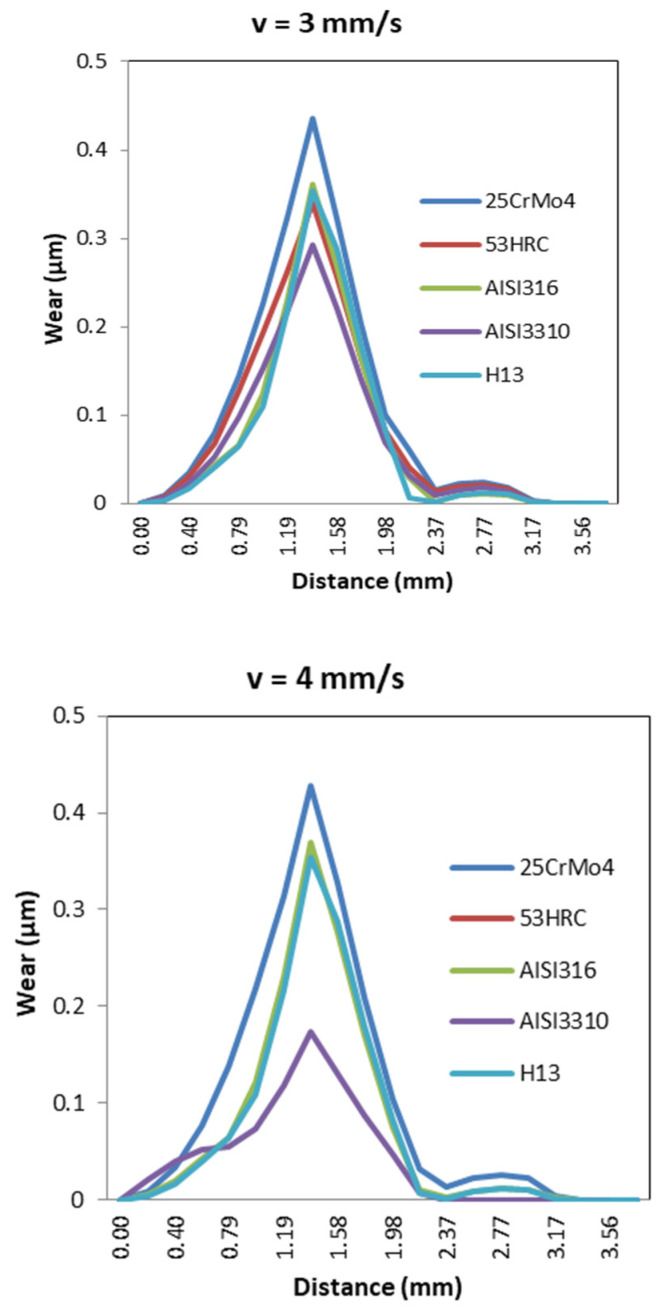
Wear distribution for high values of ram speed for different die materials.

A typical behavior in wear distribution for different die materials was characterized by the lowest wear values for ram speeds between 3 and 4 mm/s, as shown in Figure 30.

#### 3.3.2. Temperature

**Figure 31:**
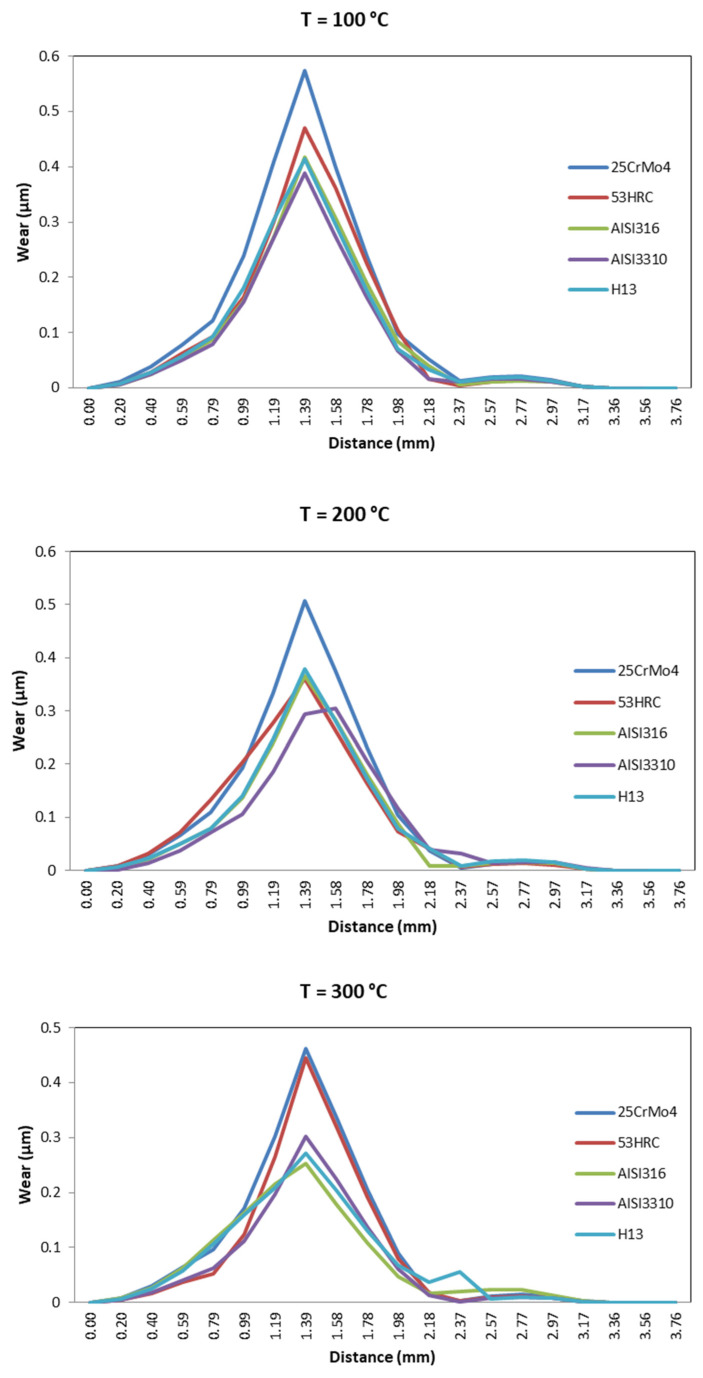
Wear distribution as a function of ram speed for different die materials.

Figure 31 shows the effect of temperature. For most die materials considered in this study, the wear decreased as the temperature increased.

**Figure 32:**
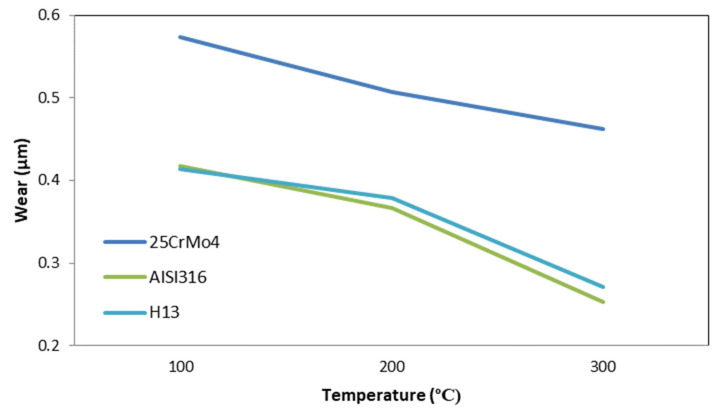
Wear evolution with temperature variation for 25CrMo4, AISI316, and H13.

As mentioned above, the general behavior is that wear decreased with the increment in temperature, but this was not the same for all the die materials, as can be observed for 53HRC. There were also die materials more sensible to the temperature rise, such as AISI316 and H13, which suffered more wear than the softer AISI3310; however, the tendency changed for high temperatures, with the materials with less wear during the process being AISI316 and H13. Figure 32 shows the general behavior of wear decreasing with the increase in temperature; only three materials are presented because the wear increased for temperatures higher than 200 °C for 53HRC, whereas the wear remained constant from 200 °C for AISI3310.

#### 3.3.3. Friction

**Figure 33:**
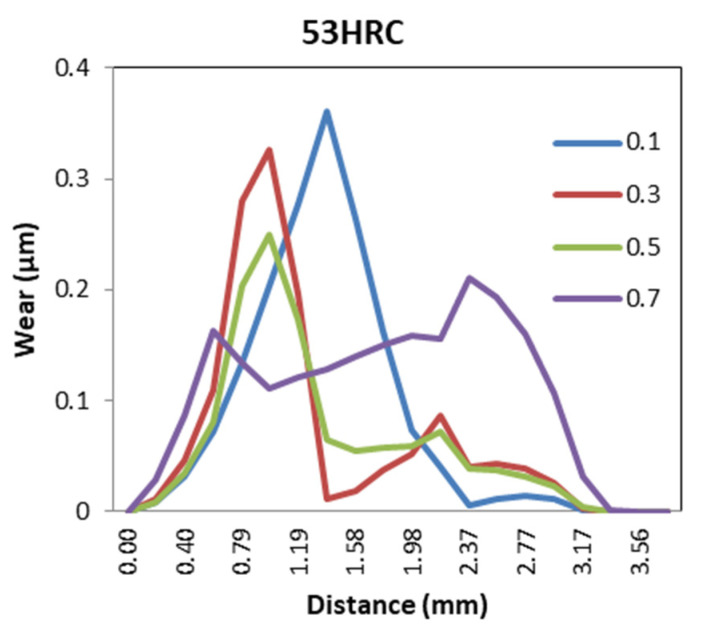

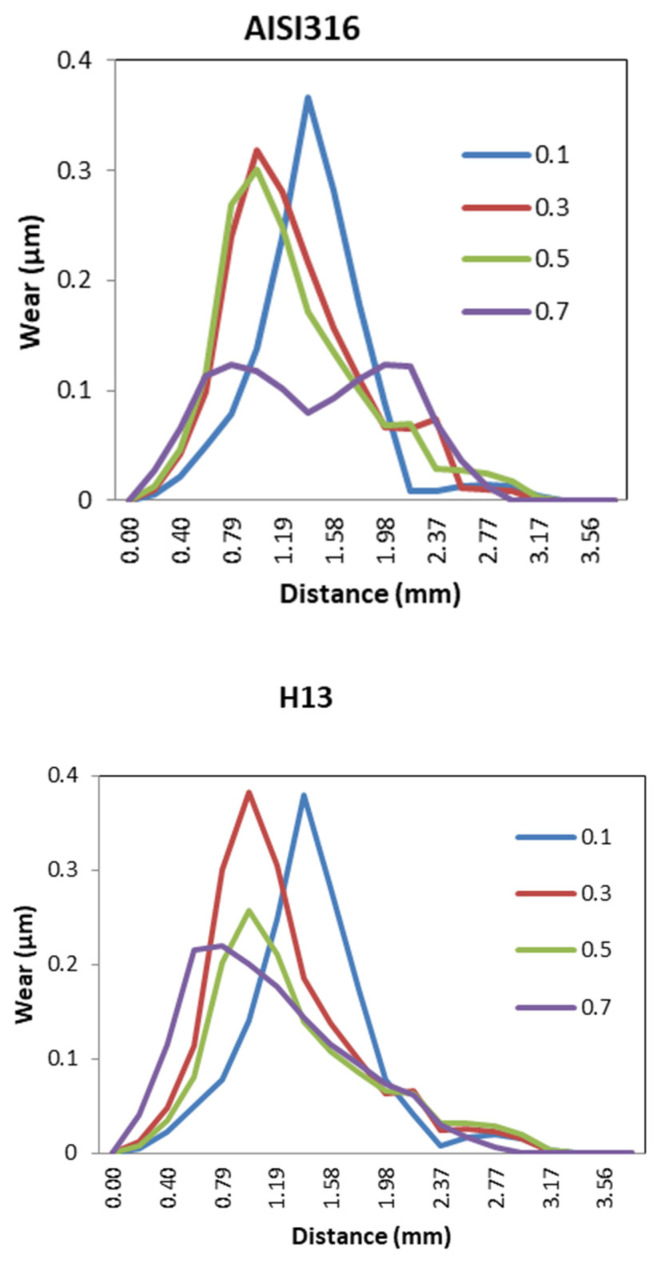
Wear distribution function of friction coefficients for 53HRC, AISI316, and H13.

The effect of the friction was inversely proportional to the wear distribution in the die. In Figure 33, it can be observed that, as friction increased, the wear distribution decreased. This was due to the reduction in sliding velocity and the increase in temperature produced by increased friction between billet and die.

For 25CrMo4 and AISI3310, there were critical values of friction coefficient (0.7 and 0.5, respectively) for which wear exceeded 1 mm. Thus, these dies could only be used once due to the geometrical tolerance of the extrudate.

## 4. Conclusions

In general, the results obtained in this paper are consistent with experimental findings and previous numerical studies reported in the literature [19,42,43,44,45]. Furthermore, experimental studies are planned to validate this numerical approach and provide better insight into the process.

According to the results, it can be concluded that the material of the die is a relevant factor to take into account when defining a multi-material coextrusion process as it has an impact on the effect of process parameters on the extrusion force required, as well as on the distribution of extrudate damage and die wear.

The die semi-angle was the parameter most affected by the die material regarding the extrusion force. Depending on the material chosen, the behavior varied with an effect on the optimum die semi-angle.

The highest difference in the extrusion force value in the same conditions for different die materials occurred at a ram speed of 1 mm/s, where the increment in extrusion force using AISI3310 was 27.50% higher than that needed for AISI316.

For the remaining parameters, the extrusion force varied between 8% and 15% of the value of the parameter requiring the lowest force.

Regarding the damage, in most configurations, the core only showed damage in the bottom part of the extrudate, in the region outside the contour of the sleeve which can be easily removed.

The damage distribution, in terms of its behavior and local values, was very similar for the different die materials; nevertheless, there were some critical values for some of the die materials where the damage was spread across the core, being a potential cause of future failure of the final part, e.g., for high values of die semi-angle (≥75°), extrusion relation (2.25), and billet height (≥30 mm). All these values, as well as their distribution across the core, were dependent of the material of the die.

The ring part was the most affected by damage, being distributed cross its entire surface (both inner and outer face). The effect of different process parameters on ring damage was very similar for all die materials studied, and there were only slightly variations in the maximum values reached.

Parameters such as temperature and ram speed had no influence on the damage.

Regarding wear distribution in the die, only three parameters were taken into account: ram speed, temperature and friction. Hardness was the most relevant parameter of the die material, whereby 25CrMo4 with the lowest hardness had the highest values of wear at the end of the process.

For 25CrMo4 and AISI3310, there were critical values of the friction coefficient at which the wear increased too much for the die to be reused.

Although the effect of these parameters was dependent of the die material, some general rules of behavior can be extracted from the results:

- Ram speed only affects the wear distribution by decreasing it for values higher than or equal to 3 mm/s.
- An increase in temperature implies a reduction in the wear.
- An increase in friction leads to a reduction in wear and a peak shift to the left (i.e., closer than point 1 in Figure 33).

In summary, it can be concluded that, to obtain an optimum coextrusion process for a bimetallic billet, it is necessary to take into account the die material and a proper combination of process parameters to achieve a balance among minimum extrusion force, minimum damage, and wear distribution.

Lastly, taking into account the results obtained in this study, improvements in the model will be implemented to incorporate the microstructure of the die materials to obtain better relationships between the selection of the die material and the extrusion force and damage. Furthermore, future lines of investigation can be opened, such as reducing the deformation load of titanium alloy due to work under relatively low temperatures or the effect of process parameters on the microstructure on the extrudate.

## Figures and Tables

**Table 1:** Chemical composition of die steels.

Material	C (wt.%)	Mn (wt.%)	Si (wt.%)	P (wt.%)	S (wt.%)	Cr (wt.%)	Mo (wt.%)	Ni (wt.%)	N (wt.%)
AISI316	0.08	2	0.75	0–0.045	0.03	16–18	2–3	10–14	0.10
H13	0.32–0.45	0.2–0.5	0.80–1.20	-	-	4.75–5.50	1.10–1.75	0.30 max	-
25CrMo4	0.22–0.29	0.60–0.90	0.10–0.40	-	-	0.90–1.20	0.15–0.30	-	-
AISI3310	0.1	0.45	0.26	-	-	1.51	0.06	3.39	-
53HRC	0.99	0.39	0.16	-	-	1.4	-	1.4	-

**Table 2:** Physical and mechanical properties of die steels.

Property	AISI316	H13	25CrMo4	AISI3310	53HRC
Density (g/cm^3^)	8.03	7.78	7.85	7.83	7.81
Tensile strength (MPa)	550	1990	670	992	1866
Yield strength (MPa)	240	1650	435	579	1800
Elastic modulus (GPa)	210	210	205	200	210
Poisson’s ratio	0.3	0.3	0.3	0.3	0.3

**Table 3:** Range of extrusion parameters.

Extrusion Parameters	Level					
	1	2	3	4	5	6
Die semi-angle (°)	15	30	45	60	75	90
Billet height (mm)	15	20	25	30	35	-
Core diameter (mm)	2	4	6	8	10	-
Ram speed (mm/s)	1	2	3	4	-	-
Friction	0.1	0.3	0.5	0.7	-	-
Temperature (°C)	100	200	300	-	-	-
Extrusion ratio	1.44	1.78	2.25	-	-	-

**Table 4:** Values of reference (baseline) for extrusion parameters.

	Die Semi-Angle (°)	Billet Height (mm)	Core Diameter (mm)	Ram Speed (mm/s)	Friction	Temperature (°C)	Extrusion Ratio
Baseline	30	20	6	2	0.1	200	1.78

## Data Availability

Not applicable.
